# The Impact of AI-Driven Chatbot Assistance on Protocol Development and Clinical Research Engagement: An Implementation Report

**DOI:** 10.3390/jpm15070269

**Published:** 2025-06-24

**Authors:** Kusal Weerasinghe, David B. Olawade, Jennifer Teke, Maines Msiska, Stergios Boussios

**Affiliations:** 1 Department of Research and Innovation, Medway NHS Foundation Trust, Gillingham ME7 5NY, UKj.teke@nhs.net (J.T.); maines.msiska@nhs.net (M.M.);; 2 Department of Allied and Public Health, School of Health, Sport and Bioscience, University of East London, London E16 2RD, UK; 3 Department of Public Health, York St John University, London E14 2BA, UK; 4 Faculty of Medicine, Health and Social Care, Canterbury Christ Church University, Canterbury CT1 1QU, UK; 5 Faculty of Life Sciences & Medicine, School of Cancer & Pharmaceutical Sciences, King’s College London, Strand, London WC2R 2LS, UK; 6 Department of Medical Oncology, Medway NHS Foundation Trust, Gillingham ME7 5NY, UK; 7 Faculty of Medicine, School of Health Sciences, University of Ioannina, 45110 Ioannina, Greece; 8 Department of Medical Oncology, Ioannina University Hospital, 45500 Ioannina, Greece; 9 AELIA Organization, 9th Km Thessaloniki—Thermi, 57001 Thessaloniki, Greece

**Keywords:** AI-driven research, chatbot assistance, clinical protocol development, healthcare innovation, research inclusivity

## Abstract

**Background:** The integration of artificial intelligence (AI) into healthcare research has the potential to enhance research capacity, streamline protocol development, and reduce barriers to engagement. Medway NHS Foundation Trust identified a plateau in homegrown research participation, particularly among clinicians with limited research experience. A generative AI-driven chatbot was introduced to assist researchers in protocol development by providing step-by-step guidance, prompting ethical and scientific considerations, and offering immediate feedback. **Methods:** The chatbot was developed using OpenAI’s GPT-3.5 architecture, customised with domain-specific training based on Trust guidelines, Health Research Authority (HRA) requirements, and Integrated Research Application System (IRAS) submission protocols. It was deployed to guide researchers through protocol planning, ensuring compliance with ethical and scientific standards. A mixed-methods evaluation was conducted using a qualitative-dominant sequential explanatory design. Seven early adopters completed a 10-item questionnaire (5-point Likert scales), followed by eight free-flowing interviews to achieve thematic saturation. **Results:** Since its launch, the chatbot has received an overall performance rating of 8.86/10 from the seven survey respondents. Users reported increased confidence in protocol development, reduced waiting times for expert review, and improved inclusivity in research participation across professional groups. However, limitations in usage due to free-tier platform constraints were identified as a key challenge. **Conclusions:** AI-driven chatbot tools show promise in supporting research engagement in busy clinical environments. Future improvements should focus on expanding access, optimising integration, and fostering collaboration among NHS institutions to enhance research efficiency and inclusivity.

## 1. Introduction

The advancement of healthcare research is crucial for driving innovation, improving patient outcomes, and ensuring evidence-based clinical practice [1]. However, promoting a research-oriented culture within healthcare institutions presents steep challenges, particularly in empowering clinicians who are new to research with the necessary support and guidance [2]. Recent systematic reviews have highlighted that AI applications in clinical research are rapidly expanding, with particular promise in protocol development and research methodology guidance [3,4]. Developing a scientifically sound and ethically robust research protocol requires extensive expertise and time—resources that many clinicians find difficult to allocate alongside their primary clinical responsibilities [5]. As a result, many healthcare institutions struggle to sustain a steady increase in homegrown studies, despite various strategic interventions aimed at fostering research activity.

The complexity of research design and protocol development presents a major barrier for new researchers [6]. Digital health interventions, including AI-powered tools, have shown potential in addressing these barriers by providing structured guidance and reducing dependency on limited human resources [7,8]. Many aspiring researchers need extensive guidance in research methodology, ethical considerations, and regulatory frameworks, in structuring a rigorous study proposal [9]. Traditionally, research-active institutions have sought to bridge this gap by providing training workshops, one-on-one consultations, and mentoring [10]. However, while effective, such approaches are often limited by resource constraints, particularly as the number of new researchers increases [11]. The iterative nature of protocol development necessitates researchers to navigate multiple rounds of feedback, leading to delays and placing additional pressure on research support teams [12].

It is within this context that Medway NHS Foundation Trust has recognised the promotion of homegrown research as a key strategic direction in establishing itself as a leader in healthcare research. Despite intense efforts to promote research engagement, the number of homegrown studies had plateaued, indicating the presence of a bottleneck in the research development process. A root cause analysis revealed that human resource constraints in providing one-on-one guidance in developing research protocols presented an obstacle, while delays were seen to be due to extensive to-and-from communications in revising the protocols [13].

A series of discussions were held with experienced researchers who had successfully built extensive research portfolios, as well as with aspiring researchers who were attempting to develop their first studies to better understand these challenges [14]. These discussions highlight that while guidance was available, the demand for expert input far exceeded the availability. New researchers needed support at various stages of protocol development, resulting in prolonged waiting times for feedback. Additionally, despite repeated consultations, common mistakes and oversights persisted in subsequent versions of research protocols, requiring numerous re-iterations [15].

Given the need for a scalable and efficient solution, Medway NHS Foundation Trust explored the potential of using generative artificial intelligence (AI), specifically a Large Language Model (LLM), to assist researchers in developing study protocols. The introduction of AI-based guidance was proposed as a means to provide researchers with real-time support in designing their study protocols [16,17]. However, several concerns were identified, including the risk of over-reliance on AI-generated suggestions, potential ethical issues, breaches of confidential/proprietary information, and the possibility of misleading research guidance. These concerns required a cautious approach to AI integration, ensuring that the AI tool was more of a supportive tool rather than a replacement for human oversight [18,19].

To address these challenges while mitigating risks, it was decided to train an LLM specifically for the purpose of guiding researchers through the research design process, prompting them to explore salient scientific and ethical considerations [20,21]. The model was designed to prompt researchers to consider key scientific and ethical considerations at every decision point, without independently generating entire research protocols. Instead, it would function as an interactive assistant, offering tailored guidance to enhance researchers’ understanding of study design principles. Additionally, to safeguard against erroneous AI-generated suggestions, the chatbot would be involved early in the research process, assisting researchers in drafting initial protocol versions that would subsequently undergo review by senior research facilitators.

The rationale behind this report is to address the identified barriers to homegrown research engagement at Medway NHS Foundation Trust by leveraging AI-driven support mechanisms. By implementing a customised LLM to assist new researchers in developing their study protocols, the institution aims to enhance research inclusivity, streamline the early-stage protocol drafting process, and reduce the burden on research facilitators. The primary research question guiding this study was: “Does AI-assisted guidance improve the quality, efficiency, and accessibility of research protocol development among clinicians with varying levels of research experience?” The primary goal of this initiative was to empower new researchers with the basic knowledge required to compile an ethically and scientifically sound study protocol, contributing to a more equitable and self-sustaining research culture within the institution.

### Related Work

The acceptance and implementation of AI-driven chatbots in healthcare settings has been increasingly studied, providing important context for our research protocol development application. Nadarzynski et al. [22] conducted a comprehensive mixed-methods study exploring healthcare professionals’ willingness to engage with AI-led health chatbots, finding moderate acceptability (67%) among participants. Their research identified key barriers including concerns about accuracy, cyber-security, and the inability of AI systems to demonstrate empathy, while acceptability was positively correlated with perceived utility, positive attitudes, and trustworthiness. These findings highlight the importance of addressing user concerns and optimising user experience for successful AI implementation.

In the medical education context, Kaur et al. [23] explored chatbot applications during the COVID-19 pandemic, identifying five key themes: chatbot utility as clinical simulation and revision tools, differential usefulness across medical school year groups, standardisation benefits for education and assessment, and implementation challenges. Their qualitative analysis revealed that both students and staff recognised clear benefits from chatbot use in medical education, though they documented significant limitations that required careful consideration during development and deployment.

Physician perspectives on healthcare chatbots were systematically examined by Palanica et al. [24] in a cross-sectional survey of 100 US practitioners. While physicians acknowledged administrative benefits, particularly for scheduling appointments (78%), locating health clinics (76%), and providing medication information (71%), they expressed substantial concerns about chatbots’ inability to address all patient needs (76%), lack of human emotional understanding (72%), and risks associated with patient self-diagnosis (74%). These studies collectively demonstrate that while AI chatbot applications in healthcare show promise, successful implementation requires careful attention to user acceptance factors, specific use-case limitations, and the critical importance of human oversight. Our work builds upon these findings by implementing a carefully supervised AI assistant specifically designed for research protocol development in resource-constrained clinical environments.

## 2. Methods

The AI-driven research assistance solution was designed to be a collaborative effort involving multiple stakeholders. Experts in research methodology, statisticians, data science professionals, sponsor representatives, and clinicians were all engaged in the planning and implementation phases. This multidisciplinary approach was essential to ensure that the final solution met both technical and practical research needs while aligning with ethical and regulatory standards.

### 2.1. Chatbot Design and Development

The chatbot was built using AI, specifically leveraging OpenAI’s GPT-3.5 architecture through the free-tier ChatGPT service. ChatGPT is a variant of the Generative Pre-trained Transformer (GPT) models, designed to generate human-like text responses based on input queries. The model, pre-trained on a broad dataset, was further fine-tuned with specific datasets to enhance its ability to provide contextually relevant responses tailored to research protocol development.

### 2.2. Domain-Specific Training

To ensure the chatbot could provide accurate and relevant guidance, a domain-specific training approach was adopted. Trust guidelines and standard templates used in protocol development (including the trust’s Research Protocol Template, Standard Operating Procedures for research governance, and Clinical Research Network guidelines) were reviewed to identify critical considerations for different research methodologies (quantitative, qualitative, mixed-methods, and pilot studies). Publicly available Health Research Authority (HRA) guidelines and questions used in the Integrated Research Application System (IRAS) portal were also analysed to compile essential ethical and scientific factors. The compiled list was then validated through discussions with research facilitators to ensure that no critical aspects had been overlooked. Further scrutiny was carried out through consultations with clinicians, research practitioners, and facilitators responsible for reviewing homegrown study protocols within the trust. These discussions refined the chatbot’s ability to prompt users to address key scientific and ethical considerations at each stage of research planning.

A flowchart was designed to outline the structured guidance provided by the chatbot. The process was mapped according to different research methodologies, ensuring that researchers were prompted to consider relevant ethical and scientific aspects at each stage. The chatbot’s behavioural instructions were customised to fit the constraints of the model’s training length, which led to the development of three sequential chatbots that guided researchers from the initiation of protocol development to the completion of the methods section.

### 2.3. Alpha-Testing

The chatbot was pretested using a range of case scenarios that included researchers with varying levels of experience (novice researchers with <1 year experience, intermediate researchers with 1–5 years experience, and experienced researchers with >5 years experience), from those proficient in research design to those with minimal prior exposure. The conversations were systematically analysed by experts in research methodology to assess the accuracy of responses, the extent to which ethical considerations were incorporated, and the logical progression of guidance. Following this testing phase, adjustments were made to the chatbot’s behavioural instructions to prevent recurring errors.

### 2.4. Risk Mitigation

One of the key challenges in designing the AI-driven research assistant was ensuring that it did not take over the research design process, which could lead to ethical concerns. To prevent this, the chatbot was programmed to serve strictly as a prompting tool, ensuring that the researcher remained the primary decision-maker at every stage. The chatbot’s role was limited to guiding users by providing relevant ethical and scientific considerations, ensuring that human judgement and expertise remained central to the research planning process.

Concerns regarding the accuracy of AI-generated content were also addressed. Despite extensive training and customisation, there remained a risk that the chatbot could produce incorrect or misleading information. This risk was mitigated by integrating the chatbot early in the research development process, ensuring that all AI-assisted drafts were subsequently reviewed by expert reviewers within the trust. The review process allowed facilitators to identify and address any inaccuracies before the protocol was finalised. The researcher was then required to revise the protocol based on feedback before receiving a letter of “Sponsorship in Principle” from the Research and Innovation (R&I) department.

### 2.5. Ethics Approval and Data Protection

The evaluation of the tool was registered with the trust’s Quality Improvement Unit as a service evaluation/usability audit that involved only NHS staff completing an anonymous online questionnaire about their experience of an in-house digital tool. No patient data were collected, no change was made to clinical care, and the findings were intended solely to inform local process improvement. Under the Health Research Authority (HRA) decision tool, this work therefore falls outside the remit of “research” that requires Research Ethics Committee (REC) review and Integrated Research Application System (IRAS) approval. All user data was anonymised, and no personally identifiable information was stored or processed.

### 2.6. Evaluation Metrics

A structured questionnaire was designed to assess the user experience with the chatbot. Consultative discussions with stakeholders were conducted to determine key evaluation criteria, which included functionality (defined as the chatbot’s ability to provide relevant prompts), usability (ease of interaction and navigation), impact (perceived improvement in protocol development confidence), and overall satisfaction (general user experience rating). Questions were formulated to specifically evaluate each of these domains while ensuring the content validity of the evaluation tool.

The complete 10-item questionnaire included the following questions using 5-point Likert scales (1 = Strongly Disagree, 5 = Strongly Agree):

1. The chatbot met my expectations for research protocol guidance.
2. The chatbot was easy to interact with and navigate.
3. The responses provided were clear and easy to understand.
4. The suggestions offered were relevant to my research needs.
5. The chatbot helped improve my confidence in protocol development.
6. I would recommend this tool to fellow researchers (1 = Extremely Unlikely, 10 = Extremely Likely).
7. The step-by-step guidance was helpful throughout the process.
8. The chatbot addressed key ethical considerations adequately.
9. The tool saved time compared to traditional consultation methods.
10. Overall, I am satisfied with the chatbot’s performance (1 = Extremely Dissatisfied, 10 = Extremely Satisfied).

The chatbot evaluation was structured to assess each of the three sequential chatbots separately before evaluating the tool as a whole. The questionnaire was pretested among a group of clinical healthcare workers to identify any ambiguities or misinterpretations in the questions. Corrections were made based on user feedback, and further refinements were introduced to enhance clarity. A pilot test was conducted with an initial group of five respondents before wider dissemination among researchers who were introduced to the chatbot tool. The audit was then registered with the Quality Improvement Unit of the trust for systematic assessment and review.

### 2.7. Study Design and Sample Size Rationale

The large language model (LLM) assistant we evaluated has no direct antecedent. Therefore, our overriding goal was surfacing unforeseen benefits, hazards and workflow frictions rather than statistical generalisation. In order to accomplish that, we adopted a qualitative-dominant sequential explanatory mixed-methods design featuring brief, informal, unstructured interviews following a survey conducted on a small sample. Seven early adopters completed the 10-item questionnaire (5-point Likert scales) that yielded headline usability metrics. To enrich and contextualise these findings, we then conducted eight free-flowing conversations to allow participants to “think aloud” to raise issues a scripted guide might never touch. These proved sufficient for thematic saturation. Expanding the quantitative arm would have yielded diminishing returns, whereas expanding the conversational arm ensured the nuanced, context-rich insights needed to refine both the UI and the underlying prompt engineering before a larger-scale trial.

## 3. Results

**Figure 1:**
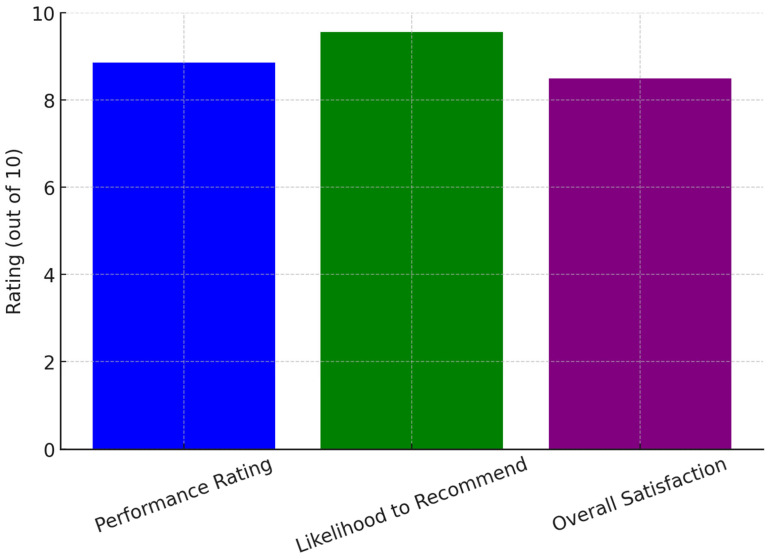
User satisfaction ratings.

Since its launch on 5 September 2024, the chatbot tool has received an overall performance rating of 8.86 out of 10 from seven survey respondents. All users who interacted with the tool agreed that it met their expectations and expressed willingness to recommend it to fellow researchers. The likelihood of a recommendation was rated at 9.57 out of 10, where 1 indicated extreme unlikelihood and 10 indicated extreme likelihood to refer the tool to others. Users unanimously agreed that the chatbot system was easy to interact with and provided clear, structured instructions throughout the research protocol development process. They also found the responses easy to comprehend, with suggestions that were both relevant and useful. Satisfaction levels were notably high, with all users expressing overall satisfaction with the chatbot’s performance. Among the seven respondents, 57% (*n* = 4) reported being extremely satisfied with the experience. Figure 1 illustrates user ratings for the AI chatbot, showing high scores in performance, likelihood to recommend, and overall satisfaction, indicating strong user approval and effectiveness in research support.

### 3.1. Respondent Demographics

Due to the small sample size, detailed demographic data that could compromise the anonymity of the participants as they could increase the risk of re-identification, including the exact role, experience levels, and the setting, were not captured. This was important in the initial survey round, as any compromise in anonymity would have potentially resulted in a bias where the agency responsible for designing and deploying the tool was directly involved in evaluation. However, as detailed in Table 1, general categories showed that professionals from various fields engaged with the chatbot, including physicians (*n* = 3), nurses (*n* = 2), senior research practitioners (*n* = 1), and allied health professionals (*n* = 1). Among those who provided feedback, 71% (*n* = 5) were affiliated with Medway NHS Foundation Trust, while 29% (*n* = 2) were external researchers. Their experience in research varied, ranging from less than one year to 14 years.

### 3.2. Qualitative Feedback

User feedback collected through the eight structured interviews highlighted the chatbot’s effectiveness in providing immediate, accessible support throughout the research planning process. A recurring theme was the sense of empowerment that users experienced due to the chatbot’s structured step-by-step guidance in compiling research protocols. This was particularly evident among early-career researchers who reported that the chatbot helped demystify the research process, making it more approachable and manageable. Many of these users noted that the knowledge acquired from interacting with the chatbot could be applied to future research endeavours, fostering increased confidence in undertaking new projects independently.

Experienced researchers, on the other hand, appreciated the chatbot’s ability to function as an interactive template that ensured all critical points in research design were addressed. A key benefit cited by this group was the potential for time savings, which was particularly valuable given their need to balance research responsibilities with demanding clinical workloads. The chatbot’s structured prompts allowed them to efficiently refine their protocols without overlooking essential components, ultimately streamlining the planning process.

Despite its positive reception, a notable drawback identified by regular users was the constraint on extended conversations due to limitations associated with the free version of ChatGPT. Some users expressed frustration over the imposed usage limits, particularly when they were working under tight deadlines. They noted that having to wait several hours after reaching the prompt limit disrupted their workflow and, at times, delayed their progress in protocol development.

### 3.3. Limitations and Self-Selection Bias

It is important to acknowledge potential self-selection bias in our results, as only users who engaged with the chatbot and chose to participate in the evaluation provided feedback. This may have resulted in more positive ratings than would be observed in a mandatory evaluation. Additionally, the short evaluation period limits our ability to assess long-term effectiveness and user retention.

## 4. Discussion

The integration of AI-driven assistance in research protocol development through the chatbot demonstrated significant benefits, particularly for clinicians new to research [25]. The results from our study highlight the chatbot’s potential in addressing long-standing barriers to research engagement, including access to timely guidance, confidence-building, and inclusivity in the research process. One of the most notable successes of the chatbot was instant feedback, which significantly improved workflow efficiency for researchers, as noted in previous findings [26,27]. Traditionally, new researchers often rely on expert mentors or research facilitators, leading to delays due to the high demand for their input [6]. This challenge has been widely documented in healthcare research, where the limited availability of research mentors has slowed down study development [28]. By providing immediate guidance, the chatbot eliminated bottlenecks and reduced waiting times, allowing researchers to progress with protocol development independently.

Another key benefit was the empowerment of researchers, especially those with limited prior experience in clinical research. Research mentorship studies have shown that early-career researchers benefit significantly from structured, scaffolded learning experiences [9,10]. The chatbot played a similar role, offering a structured step-by-step process while prompting researchers to think critically about ethical and scientific considerations [29,30]. By limiting its function to offering guidance rather than designing research protocols independently, the chatbot ensured that researchers retained agency in the research process. This approach aligns with recommendations from AI ethics literature, which emphasises the importance of human oversight in AI-assisted decision making [19,31].

Our designed chatbot also contributed to inclusivity in homegrown research, broadening participation beyond experienced researchers. Traditionally, clinical research studies were predominantly designed by physicians, while other healthcare professionals such as nurses and allied health professionals have had lower engagement in designing research projects. Our chatbot facilitated a more balanced research landscape by providing accessible guidance to these underrepresented groups, a concern noted in recent literature [32,33]. Data from Medway NHS Foundation Trust showed that nearly one-third of homegrown research was led by non-physician researchers after the chatbot’s introduction, suggesting a shift towards a more inclusive research culture.

### 4.1. AI Biases and Validation Challenges

Despite these advantages, several limitations merit discussion. AI biases represent a significant concern in any LLM application [34]. During our validation process, we identified instances where the chatbot provided responses that reflected biases present in its training data, particularly regarding research methodologies favoured in high-income settings. To address this, our expert review process specifically examined AI-generated suggestions for cultural and methodological biases, with facilitators trained to identify and correct such issues before protocol finalisation.

The usage constraints imposed by OpenAI’s free-tier access presented a significant challenge [35]. Many researchers relied on the free version, which limited conversation length and required a cooling-off period before resuming interactions. This challenge disproportionately affected novice researchers who needed longer conversations to refine their protocols. Existing research on AI accessibility in professional settings has highlighted similar concerns, with studies noting that limited AI access hinders workflow continuity and user experience [12,13]. While these constraints did not impact most clinicians who could only dedicate short bursts of time to research, those working under tight deadlines found it disruptive to their progress.

### 4.2. Cost–Benefit Analysis and Scalability

The cost–benefit trade-offs of different implementation approaches require careful consideration. ChatGPT Enterprise would cost approximately GBP 20 per user per month, which for a trust with 200 potential research users would represent GBP 48,000 annually. API-based integration offers more flexible pricing at approximately GBP 0.002 per 1000 tokens, potentially reducing costs for light users but requiring significant upfront IT infrastructure investment. Open-source LLMs like Llama 2 would eliminate licencing costs but require substantial technical expertise and computational resources, with estimated development costs of GBP 50,000–100,000 and ongoing maintenance requirements [36].

### 4.3. Generalisability to Resource-Constrained Settings

While our findings are promising within the NHS context, their applicability to low-resource settings requires consideration. The chatbot’s reliance on stable internet connectivity and English-language prompts may limit its utility in settings with infrastructure constraints or non-English speaking populations. However, the core principle of AI-assisted research guidance could be adapted with appropriate localisation and infrastructure support [37].

## 5. Future Direction and Recommendations

Given the overwhelmingly positive feedback from users and the challenges posed by AI access limitations, several solutions have been explored to enhance the chatbot’s effectiveness. After discussions with subject matter experts and stakeholders, three primary options were considered for overcoming the identified obstacles:

### 5.1. Short-Term Implementation Strategy

Implementing API-based integration with Medway’s intranet as an immediate measure would ensure the continuity of chatbot usage while addressing accessibility issues. A pilot phase with a limited user group can be launched to evaluate effectiveness and cost efficiency.

### 5.2. Long-Term Strategic Planning

Exploring the feasibility of adopting ChatGPT Enterprise should be prioritised in discussions with hospital administration, balancing cost considerations with research productivity benefits. In parallel, feasibility studies for developing a custom chatbot using open-source LLMs should be initiated to determine whether this is a viable long-term alternative.

### 5.3. Capacity Building and Training

Training sessions should be conducted for researchers to optimise chatbot usage. Providing guidance on structuring interactions efficiently could mitigate some of the limitations associated with free-tier usage in the interim.

### 5.4. Monitoring and Evaluation Framework

Continuous assessment of chatbot effectiveness through periodic feedback collection will help refine the tool’s functionality and ensure alignment with evolving research needs. Establishing a dedicated oversight team to manage AI research support initiatives would further enhance sustainability.

### 5.5. NHS-Wide Adoption Strategy

For broader NHS implementation, we recommend establishing pilot partnerships between 3 and 5 NHS trusts to evaluate cross-institutional effectiveness. This should include standardised evaluation metrics, shared training resources, and collaborative refinement of prompt engineering based on diverse institutional needs.

## 6. Strengths and Limitations of the Report

### 6.1. Strengths

One of the key strengths of this report is its comprehensive evaluation of AI-assisted research guidance. The study systematically assessed the chatbot’s impact on research accessibility, efficiency, and inclusivity, providing both quantitative and qualitative insights. The combination of structured survey data and user feedback ensures that the findings are not only statistically significant but also contextually relevant to the challenges faced by new researchers.

Another strength is the real-world applicability of the findings. Unlike theoretical discussions on AI in research, this study is grounded in actual implementation within a healthcare setting. The report provides valuable insights for institutions considering AI-based solutions to support research engagement, particularly in environments where mentorship and expert review resources are limited. The chatbot’s ability to bridge knowledge gaps and facilitate research participation among non-physician professionals further highlights its practical significance.

### 6.2. Limitations

Despite its strengths, the report has some limitations that should be acknowledged. One primary limitation is the reliance on a single AI model (GPT-3.5) for chatbot implementation. While the model was customised for research guidance, its responses remain subject to inherent biases and limitations associated with large language models. The chatbot’s knowledge is restricted to pre-existing datasets, and it may not always reflect the latest updates in research regulations or specific institutional guidelines.

Another limitation is the restricted evaluation period, as the chatbot had only been in use for approximately two months at the point of assessment. While initial findings are promising, longer-term studies are necessary to determine the sustained impact of AI-driven research assistance on protocol quality, research completion rates, and user confidence over time. Future research should incorporate longitudinal studies to assess how engagement evolves as more users adopt the tool.

## 7. Conclusions

The implementation of a generative AI-driven chatbot tool for research protocol development at Medway NHS Foundation Trust has demonstrated its effectiveness as a valuable support system for healthcare professionals, particularly those new to research. By providing structured step-by-step guidance, prompting researchers to consider key ethical and scientific elements, and offering immediate feedback, the chatbot significantly reduced the long-standing issue of waiting times for expert support. This, in turn, empowered users to develop research protocols with greater confidence and efficiency.

The strong uptake among a diverse range of healthcare professionals reflects the chatbot’s success in bridging gaps in research capacity and fostering a more inclusive research environment. Previously, homegrown research was largely physician-led, but the tool has enabled broader participation, particularly among nurses, allied health professionals, and senior management. This shift represents a meaningful step toward democratising research engagement within the trust.

Despite these successes, challenges remain. The primary limitation identified was the restricted access due to platform-imposed usage constraints, which affected researchers requiring longer conversations for protocol refinement. This underscores the need for continued investment in scalable AI solutions tailored to institutional research needs. However, the success of this chatbot initiative demonstrates the potential for LLM-driven tools to become an integral part of research and innovation strategies within busy clinical environments.

## 8. Call to Action

The positive outcomes of this initiative call for further exploration and collaboration among researchers, healthcare administrators, and technology providers. NHS organisations are encouraged to consider adopting similar AI-based tools to enhance research capacity, improve accessibility, and promote inclusivity in research. By sharing insights, experiences, and technical expertise across different NHS trusts, institutions can collectively accelerate the development and refinement of AI-driven research support tools. Addressing shared barriers through collaborative learning and technology deployment will ensure that researchers across the healthcare system have access to efficient, scalable support mechanisms.

Additionally, this initiative at Medway NHS Foundation Trust serves as a compelling example of how AI can be thoughtfully integrated into research environments while maintaining human oversight. As AI technologies continue to evolve, ongoing ethical considerations and user-centred refinements must remain at the forefront of development efforts. Ensuring that AI-driven solutions complement rather than replace human expertise will be key to sustaining trust and effectiveness in clinical research settings.

By investing in scalable, user-friendly AI tools, healthcare organisations may enable their workforce to conduct ethically sound, scientifically rigorous research, ultimately leading to evidence-based improvements in patient care. This initiative demonstrates that when properly designed and implemented, AI has the potential to transform research processes, making them more accessible, efficient, and inclusive.

## Figures and Tables

**Table 1:** Summary of survey respondents.

Characteristic	Number (%)
**Professional Role**	
Physicians	3 (43%)
Nurses	2 (29%)
Senior Research Practitioners	1 (14%)
Allied Health Professionals	1 (14%)
**Affiliation**	
Medway NHS Foundation Trust	5 (71%)
External Researchers	2 (29%)
**Research Experience**	
<1 year	2 (29%)
1–5 years	3 (43%)
>5 years	2 (29%)

## Data Availability

The original contributions presented in this study are included in the article. Further inquiries can be directed to the corresponding author.

## References

[1] Slade S., Philip K., Morris M. (2018). Frameworks for embedding a research culture in allied health practice: A rapid review. Health Res. Policy Syst..

[2] Lee S., Gifford J., Flood V. (2024). Enablers and Barriers of Research Engagement Among Clinician Researchers: Nursing, Allied Health and Medical Professionals. J. Multidiscip. Healthc..

[3] Ahuja A., Murti Y., Singh S. (2024). Exploring the potential of artificial intelligence in medical research: Applications, regulatory concerns, opportunities and future outlook-a mini review. Lett. Drug Des. Discov..

[4] Nuka S.T. (2022). The role of AI driven clinical research in medical device development: A data driven approach to regulatory compliance and quality assurance. Glob. J. Med. Case Rep..

[5] Thiga M., Kimeto P., Walekhwa M. (2024). An Integrated Framework for Scientific and Ethical Review of Research Proposals. J. Res. Acad. Writ..

[6] Matheson M., Skinner I.W., Vehagen A., Auliffe S.M., Malliaras P. (2025). Barriers and Enablers of Primary Healthcare Professionals in Health Research Engagement: A Systematic Review of Qualitative Studies. Nurs. Health Sci..

[7] Hyder A.A., Selig H., Ali J., Rutebemberwa E., Islam K., Pariyo G. (2019). Integrating capacity development during digital health research: A case study from global health. Glob. Health Action.

[8] Olawade D.B., Omeni D., Gore M.N., Hadi M. (2025). Enhancing qualitative research through virtual focus groups and artificial intelligence: A review. Int. J. Med. Inform..

[9] Sriram N. (2025). Effective Research Proposals Writing in Medical, Pharmacy, and Allied Health Sciences. Int. J. Pharm. Health Care Res..

[10] Nearing K., Nuechterlein B., Tan S., Zerzan J., Libby A., Austin G. (2020). Training Mentor-Mentee Pairs to Build a Robust Culture for Mentorship and a Pipeline of Clinical and Translational Researchers: The Colorado Mentoring Training Program. Acad. Med..

[11] Bonifacino E., Ufomata E., Farkas A., Turner R., Corbelli J. (2021). Mentorship of Underrepresented Physicians and Trainees in Academic Medicine: A Systematic Review. J. Gen. Intern. Med..

[12] Merga M., Mason S. (2021). Mentor and peer support for early career researchers sharing research with academia and beyond. Heliyon.

[13] Groeneveld B., Dekkers T., Boon B., D’Olivo P. (2018). Challenges for design researchers in healthcare. Des. Health.

[14] Sorkness C.A., Pfund C., Ofili E.O., Okuyemi K.S., Vishwanatha J.K., NRMNteam Zavala M.E., Pesavento T., Fernandez M., Tissera A., Deveci A. (2017). A new approach to mentoring for research careers: The National Research Mentoring Network. BMC Proc..

[15] Johnson M.O., Gandhi M. (2015). A mentor training program improves mentoring competency for researchers working with early-career investigators from underrepresented backgrounds. Adv. Health Sci. Educ..

[16] Brannan G.D., Dumsha J.Z., Yens D.P. (2013). A research primer: Basic guidelines for the novice researcher. J. Osteopath. Med..

[17] Munagandla V.B., Dandyala S.S., Vadde B.C., Engineer D. (2024). AI-Driven Optimization of Research Proposal Systems in Higher Education. Rev. Intelig. Artif. Med..

[18] Khalifa M., Albadawy M. (2024). Using artificial intelligence in academic writing and research: An essential productivity tool. Comput. Methods Programs Biomed. Update.

[19] Resnik D.B., Hosseini M. (2024). The ethics of using artificial intelligence in scientific research: New guidance needed for a new tool. AI Ethics.

[20] Chen Z., Chen C., Yang G., He X., Chi X., Zeng Z., Chen X. (2024). Research integrity in the era of artificial intelligence: Challenges and responses. Medicine.

[21] Filetti S., Fenza G., Gallo A. (2024). Research design and writing of scholarly articles: New artificial intelligence tools available for researchers. Endocrine.

[22] Nadarzynski T., Miles O., Cowie A., Ridge D. (2019). Acceptability of artificial intelligence (AI)-led chatbot services in healthcare: A mixed-methods study. Digit. Health.

[23] Kaur A., Singh S., Chandan J.S., Robbins T., Patel V. (2021). Qualitative exploration of digital chatbot use in medical education: A pilot study. Digit. Health.

[24] Palanica A., Flaschner P., Thommandram A., Li M., Fossat Y. (2019). Physicians’ perceptions of chatbots in health care: Cross-sectional web-based survey. J. Med. Internet Res..

[25] Bali J., Garg R., Bali R.T. (2019). Artificial intelligence (AI) in healthcare and biomedical research: Why a strong computational/AI bioethics framework is required?. Indian J. Ophthalmol..

[26] Aithal P.S., Aithal S. (2023). Optimizing the use of artificial intelligence-powered GPTs as teaching and research assistants by professors in higher education institutions: A study on smart utilization. Int. J. Manag. Technol. Soc. Sci..

[27] Ransdell L., Wayment H., Schwartz A., Lane T., Baldwin J. (2021). Precision mentoring (PM): A proposed framework for increasing research capacity in health-related disciplines. Med. Educ. Online..

[28] Nunez M., Sakuma Y., Conyers H., Day S., Terris-Prestholt F., Ong J.J., Pan S.W., Shakespeare T., Tucker J.D., Kpokiri E.E. (2024). Health research mentorship in low-income and middle-income countries: A global qualitative evidence synthesis of data from a crowdsourcing open call and scoping review. BMJ Glob. Health.

[29] Opara E., Mfon-Ette Theresa A., Aduke T.C. (2023). ChatGPT for teaching, learning and research: Prospects and challenges. Glob. Acad. J. Humanit. Soc. Sci..

[30] Ghorashi N., Ismail A., Ghosh P., Sidawy A., Javan R., Ghorashi N.S. (2023). AI-powered chatbots in medical education: Potential applications and implications. Cureus.

[31] Bhattamisra S.K., Banerjee P., Gupta P., Mayuren J., Patra S., Candasamy M. (2023). Artificial intelligence in pharmaceutical and healthcare research. Big Data Cogn. Comput..

[32] Rubinger L., Gazendam A., Ekhtiari S., Bhandari M. (2023). Machine learning and artificial intelligence in research and healthcare. Injury.

[33] Nadarzynski T., Knights N., Husbands D., Graham C.A., Llewellyn C.D., Buchanan T., Montgomery I., Ridge D. (2024). Achieving health equity through conversational AI: A roadmap for design and implementation of inclusive chatbots in healthcare. PLOS Digit. Health.

[34] Huang X., Ruan W., Huang W., Jin G., Dong Y., Wu C., Bensalem S., Mu R., Qi Y., Zhao X. (2024). A survey of safety and trustworthiness of large language models through the lens of verification and validation. Artif. Intell. Rev..

[35] Purushothaman R. (2025). A comprehensive analysis of consumer behavior of OpenAI. SSRN Prepr..

[36] Thurzo A., Varga I. (2025). Revisiting the Role of Review Articles in the Age of AI-Agents: Integrating AI-Reasoning and AI-Synthesis Reshaping the Future of Scientific Publishing. Bratisl. Med. J..

[37] Borger J.G., Ng A.P., Anderton H., Ashdown G.W., Auld M., Blewitt M.E., Brown D.V., Call M.J., Collins P., Freytag S. (2023). Artificial intelligence takes center stage: Exploring the capabilities and implications of ChatGPT and other AI-assisted technologies in scientific research and education. Immunol. Cell Biol..

